# Synthesis of 2,4,6-Trinitrotoluene (TNT) Using Flow Chemistry

**DOI:** 10.3390/molecules25163586

**Published:** 2020-08-06

**Authors:** Dimitris Kyprianou, Michael Berglund, Giovanni Emma, Grzegorz Rarata, David Anderson, Gabriela Diaconu, Vassiliki Exarchou

**Affiliations:** Joint Research Centre, European Commission, Retieseweg 111, 2440 Geel, Belgium; Dimitris.Kyprianou@ec.europa.eu (D.K.); michael@berglund.be (M.B.); giovanni.emma@ec.europa.eu (G.E.); Grzegorz.RARATA@ec.europa.eu (G.R.); gabriela.diaconu@ec.europa.eu (G.D.); Vasiliki.EXARCHOU@ec.europa.eu (V.E.)

**Keywords:** nitration, TNT, flow chemistry, synthesis, explosives, explosive standards, testing

## Abstract

This paper describes the nitration of 2,4-dinitrotoluene (DNT) and its conversion to 2,4,6-trinitrotoluene (TNT) at a gram scale with the use of a fully automated flow chemistry system. The conversion of DNT to TNT traditionally requires the use of highly hazardous reagents like fuming sulfuric acid (oleum), fuming nitric acid (90–100%), and elevated temperatures. Flow chemistry offers advantages compared to conventional syntheses including a high degree of safety and simpler multistep automation. The configuration and development of this automated process based on a commercially available flow chemistry system is described. A high conversion rate (>99%) was achieved. Unlike established synthetic methods, ordinary nitrating mixture (65% HNO_3_/98% H_2_SO_4_) and shorter reaction times (10–30 min) were applied. The viability of flow nitration as a means of safe and continuous synthesis of TNT was investigated. The method was optimized using an experimental design approach, and the resulting process is safer, faster, and more efficient than previously reported TNT synthesis procedures. We compared the flow chemistry and batch approaches, including a provisional cost calculation for laboratory-scale production (a thorough economic analysis is, however, beyond the scope of this article). The method is considered fit for purpose for the safe production of high-purity explosives standards at a gram scale, which are used to verify that the performance of explosive trace detection equipment complies with EU regulatory requirements.

## 1. Introduction

The synthesis of 2,4,6-trinitrotoluene (TNT) has gained a lot of scientific and industrial interest since it was the first high explosive that was able to fulfil the expectations of producers and the military. It was first synthesized in the 1860s and was later produced in large quantities during World War I and World War II [1]. This explosive is a moderately powerful, high-energy material, with satisfactory thermal stability and reduced mechanical sensitivity. It is still used in many explosive mixtures today by military and special branches of industry. This is facilitated by its low cost and the fact that it is relatively insensitive, as well as readily melt-castable. It is, therefore, still a main component in many explosive mixtures, some of which were developed several decades ago, such as Amatol, Baratol, Comp B, H-6, Tritonal, and Torpex [2].

In recent years, considerable progress has been made in the synthesis of high-energy materials, especially in the field of military high explosives or propellants. Some of these high-energy materials can be obtained by novel eco-friendly methods of synthesis or techniques [3,4]. Nevertheless, the traditional approach is still applied, and it involves the use of hazardous concentrated acid mixtures (typically nitric and sulfuric acid as a nitrating mixture [5]). Nitration processes carried out especially at a larger scale are particularly prone to runaway exothermic reactions, and thus are of high safety concern [6].

High purity TNT can be obtained after nitration of the dinitrotoluene (DNT) isomers: 2,4-DNT and 2,6-DNT. By applying a conventional synthesis, highly concentrated nitric acid (100%) and oleum (sulfuric acid containing up to 60% SO_3_) are required to achieve a conversion rate higher than 98% as required for military grade TNT [7,8]. This way of synthesis presents safety concerns since the handling, mixing, and disposal of oleum with anhydrous nitric acid is particularly dangerous [9,10,11].

Several methods for TNT synthesis or nitration of aromatic compounds other than the traditional method are patented or reported in the literature [8]. They focus mainly on improving the process by achieving higher purity, faster reaction times, and more environmentally friendly approaches. Some examples include the methods developed by Millar et al., who performed the nitration of DNT in batch mode by using N_2_O_5_/H_2_SO_4_ 98% as the nitrating mixture, Lagoviyer et al. that used sodium nitrate/molybdenum oxide for nitration of toluene, and Kyler et al. that patented the use of 98–99% nitric acid with trifluoromethanesulfonic acid for the conversion of DNT to TNT [7,11,12].

The objective of this work was to develop a safer process for the manufacturing of high purity TNT (>99%) to be used in the preparation of explosives standards at the European Commission’s Joint Research Centre. These standards are used to verify that various explosives detection devices, like explosives trace detection equipment (ETD) used at airports, perform according to the specifications laid down in the EU Commission Implemented Regulation 2015/1998 [13]. In this regard, flow chemistry was chosen as a safer alternative to the conventional method of preparing TNT.

Flow chemistry—also known as continuous flow chemistry—is the process of performing chemical reactions in a reactor, which can be a pipe, tube, or more complex microstructure device. The reagents are pumped to a mixing junction and flow into the temperature-controlled reactor. The large surface area facilitates vigorous mixing due to high rates of mass transfer and fast dissipation of heat, which allows for highly exothermic reactions. Consequently, faster, safer, automated, scalable procedures can be developed, and high purity products can be obtained by applying this form of synthesis [14,15,16]. In the pharmaceutical sector, several highly exothermic or hazardous nitration reactions were scaled up using flow chemistry processes [17]. Energetic materials have traditionally been prepared in batch reactors. However, on some occasions, flow chemistry was successfully used as an alternative to batch synthesis [18,19]. Among explosive substances, nitroglycerin, which is also a pharmaceutical substance, attracted a significant scientific interest for translating its conventional batch synthesis into flow process [20].

The application of flow chemistry is important for processes associated with large risks. Flow chemistry mainly increases safety with well-controlled pressure, stable temperatures, homogenous mixing, and fast dissipation of heat. Moreover, lesser amounts of energetic materials are present at any time in the reactor due to the continuous flow of reagents and the removal of the synthesis products. Although flow chemistry can be beneficial, the methods can be complex to develop. The methods and reagents used must in many cases be modified in order to be compatible with a flow chemistry application.

**Figure 1:**
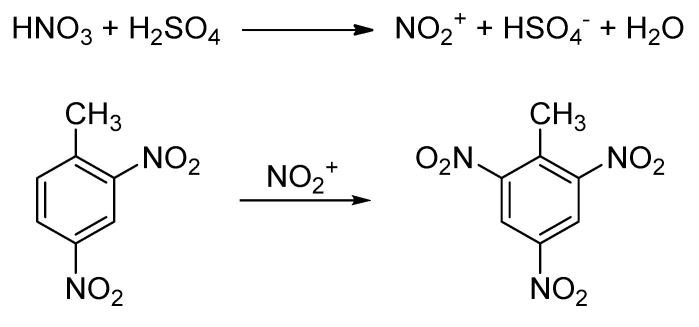
Synthetic path for the conversion of 2,4-dinitrotoluene (2,4-DNT) to 2,4,6-trinitrotoluene (TNT).

In the current work, the possibility of performing the conversion of 2,4-DNT to TNT (third nitration step of TNT synthesis) using flow chemistry and an ordinary 98% sulfuric/65% nitric acid nitrating mixture instead of oleum and anhydrous nitric acid was investigated. This reaction is an electrophilic aromatic substitution and it is depicted in Figure 1 [21].

This third step is the most challenging one in order to obtain a high conversion rate because side reactions, oxidations, or other break-down processes can also take place, leading to the formation of several by-products. Several accidents during TNT manufacturing have been reported [8] (p. 349, p. 391).

During the development of the flow chemistry method, several challenges were encountered, such as clogging due to precipitation of TNT in the outlet flow stream. After the initial method development, the main factors affecting the purity of the product were identified and optimized using a design of experiments (DoE) approach. The DoE approach presents several advantages compared to the so-called OVAT (one variable at a time) approach and flow chemistry processes are ideally suited for it [22,23]. This is because experimental parameters such as temperature, pressure, flow rate, amount of reagent, and residence time can be easily controlled and finely regulated in a fully automated system. The chromatographic purity of the synthesized products was determined using HPLC-DAD. ^1^H NMR was used to detect possible by-products and impurities in the final product.

## 2. Results and Discussion

### 2.1. Preliminary Studies

Preliminary experiments were performed in order to investigate whether it is feasible to perform the reaction in flow chemistry and if high conversion rates could be achieved. Contrary to reported flow chemistry methods for synthesis of liquid energetic materials [18,19,20], this application was particularly challenging for several reasons. The product, TNT, is a solid substance and can precipitate in the reaction mixture causing clogging. Moreover, long reaction times (usually 4–6 h) and mixtures of oleum-fuming nitric acid are normally required for obtaining military grade TNT (pp. 348–364, [8]), [10,12], and. This range of reaction time is considered too long for continuous processing due to the limitations related to the reactor volume and the minimum range of the flow rate of the pumps. In the method of TNT synthesis described by Millar et al., this step of nitration is performed in a batch mode due to the unsuitability of reagents and products in flow systems, namely acid mixtures are too viscous and there is the possibility of precipitation of flow solid product [7], (p. 13, [24]).

In our study, we found that H_2_SO_4_ 98% was the most suitable substance to be used as both the reaction solvent and reagent. A chloroform stream was also added to the outlet flow stream in the final reaction set up, as described in materials and methods. The addition of chloroform at this step prevents any precipitation of the product and clogging due to the lower solubility of TNT in the sulfuric/nitric acids mixture compared to sulfuric acid alone [6]. It should be noted that introducing chloroform in the reactor inlet flow can cause trichloronitromethane (chloropicrin) to be formed [25] (p. 252).

The use of chloroform has the following additional advantages:

1. It does not interfere with the reaction since the mixture is cooled down and the temperature is not high enough to stimulate the nitration of chloroform to the toxic chloropicrin;
2. it quenches the reaction, preventing possible oxidations from occurring; and
3. it facilitates the purification process and extraction of TNT from the nitrating mixture.

The preliminary results demonstrated the feasibility of performing the 2,4-DNT nitration in flow using the ordinary nitrating mixture (HNO_3_ 65%, H_2_SO_4_ 98%) with a satisfactory conversion rate.

### 2.2. Reaction Optimization and Experimental Design

Based on the preliminary experiments and reported literature, the HNO_3_:DNT molar ratio, residence time (or reaction time), and temperature of the reaction were identified as the key parameters that have a significant impact on the conversion rate of 2,4-DNT to TNT [8] (p. 84). The ranges of these parameters fed into the DoE software (MODDE) for flow synthesis optimization were: HNO_3_:DNT molar ratio between 5:1 and 1:1, residence time 10–30 min, and temperature 110–150 °C. The application of the DoE software is considered very important for assessing the parameters that have a significant impact on the conversion rate and for determining the best reaction conditions by performing the minimal number of experiments. The range of the parameters applied during the experimental design were determined according to observations in the preliminary experiments. The DoE method applied is described in Section 3.2.1. The experiments performed and the conversion rates obtained are reported in Table 1.

As illustrated in Table 1, seven experiments yielded highly pure TNT (i.e., ≥99.0%, as determined by HPLC-DAD). The estimated conversion rates were calculated after HPLC analysis of the solid product obtained after extraction as described in Section 3.2.2 and Section 3.2.4. The isolated yield after the extraction step was 58–70% of the amount expected according to the DNT moles fed (pumped) into the reactor. The lower value of the isolated yield compared to the industrial methods or patents reported in the literature (80–92%) can be attributed to the solubility of TNT in the mineral acid aqueous phase, which results in significant losses during the extraction process [8] (pp. 292–294). Further optimization of the extraction process was not in the scope of the current study.

**Figure 2:**
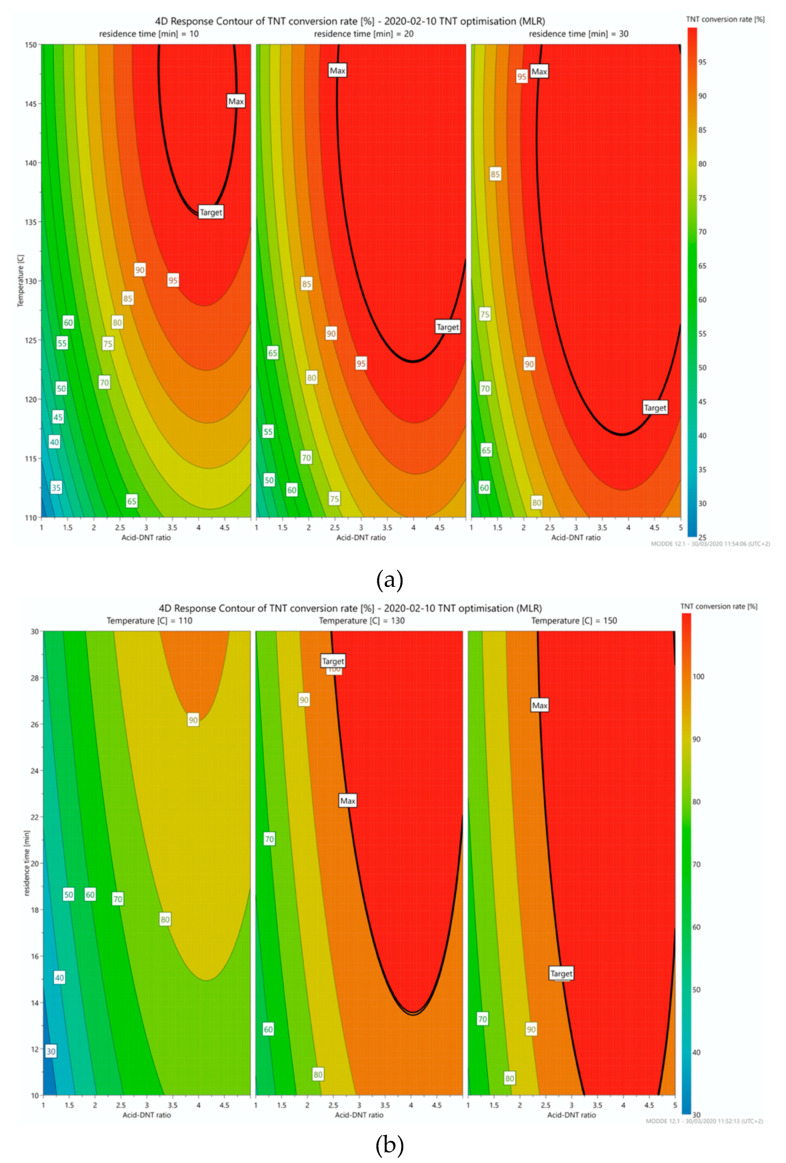
(**a**) Conversion rates in relation with temperature and HNO_3_:DNT molar ratio at 10, 20, and 30 min residence time and (**b**) conversion rates in relation with residence time and HNO_3_:DNT molar ratio at 110, 130, and 150 °C.

In order to better assess the impact of the investigated parameters on the conversion rate and to identify the range of the reaction conditions resulting in a high conversion rate (>99%), as determined by HPLC-DAD, contour plots were obtained from the MODDE software. Figure 2 shows the effect of reaction temperature (°C) and HNO_3_:DNT molar ratio on the conversion rate at 10, 20, and 30 min residence time and the effect of residence time and HNO_3_:DNT molar ratio on the conversion rate at 110, 130, and 150 °C.

As illustrated in Figure 2a,b and Table 1, the three factors studied had a significant impact on the conversion rate. The effect of these factors and the conditions resulting in high product purity will be discussed in detail.

#### 2.2.1. Effect of HNO_3_:DNT Molar Ratio

As shown in Figure 2, HNO_3_ should be in excess for achieving high conversion rates. Nevertheless, higher temperature and residence time result in lower values of the HNO_3_:DNT molar ratio needed to achieve high purity TNT. In the range of the applied reaction conditions, an excess of HNO_3_ > 2.5 is required to obtain a high purity product. This conclusion is in agreement with other reported methods where an excess of nitric acid is suggested for a faster and complete nitration of DNT [7,8,10,12]. Moreover, excess nitric acid during DNT nitration is important, especially at high temperatures, in order to prevent decomposition reactions from occurring [8] (p. 78)

#### 2.2.2. Effect of Residence Time

In order to complete the nitration of 2,4-DNT at a short residence time, a higher temperature and HNO_3_:DNT molar ratio should be used. This is clearly shown in Figure 2a in which the area of high purity (red) is observed on the top right corner of the contour plot. As an example, to obtain a conversion rate >99% with 10 min residence time, 150 °C and a molar ratio HNO_3_:DNT = 5 should be applied (Section 2.2, experiment number 4 in Table 1). By observing the three graphs in Figure 2a, it can be concluded that with longer residence time, lower reaction temperatures are needed to observe a satisfactory conversion rate (>99%).

#### 2.2.3. Effect of Temperature

Temperature of the reaction appears to be a key factor in order to achieve the aimed conversion rates. As shown in Figure 2b and Table 1, at 110 °C it was not possible to achieve the target purity level of TNT within the range of parameters investigated in this study. It is predicted by the DoE model (Figure 2a) that the minimum temperatures required to achieve the target purity are around 137, 125, and 117 °C for residence times of 10, 20, and 30 min, respectively. These predictions are in close agreement with our observations during preliminary experiments. In the second and third graph of Figure 2b, the combinations of HNO_3_:DNT molar ratio and residence times that yield the desired purity TNT (>99%) at 130 and 150 °C, are illustrated. As better highlighted in the third graph, the target area is the dominant part of the contour plot, meaning that for higher temperatures, lower residence times are necessary.

### 2.3. Batch vs. Flow Synthesis

**Figure 3:**
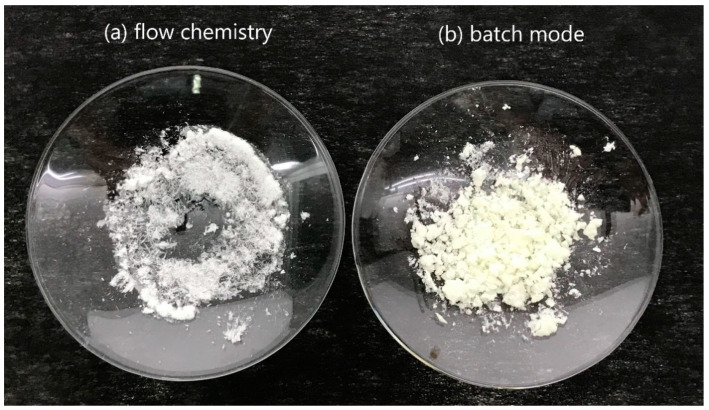
TNT produced from (**a**) flow chemistry and (**b**) batch mode. The flow chemistry sample is white, whereas the batch mode sample has a yellow hue caused by impurities.

**Figure 4:**
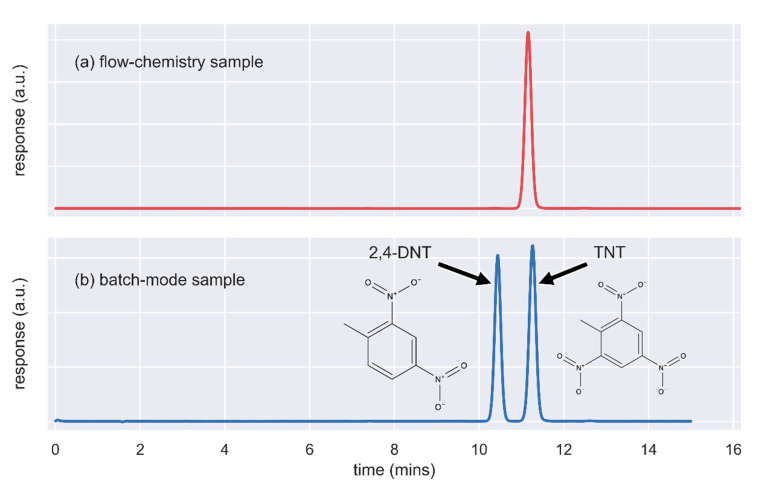
Chromatograms of HPLC-DAD analysis of the product obtained after 2,4-DNT nitration using (**a**) flow chemistry and (**b**) batch mode.

In order to demonstrate the difference of the reaction rate for DNT nitration in flow and batch synthesis, a comparative study was conducted. Therefore, the optimum reaction conditions used in flow chemistry were applied for a batch-type reaction (HNO_3_ 65%:H_2_SO_4_ 98% = 3:1, 130 °C, 20 min). It should be mentioned that according to our knowledge, no scientific data are available regarding DNT nitration with these conditions. The conversion in batch mode did not exceed 58% due to the use of a different nitrating mixture (HNO_3_ 65%:H_2_SO_4_ 98% instead of HNO_3_ 100%/oleum) and much shorter reaction time. For these reasons, the obtained result was expected as the conditions applied for batch synthesis were not optimized. Images of the TNT produced from flow chemistry and from batch mode are shown in Figure 3a,b, respectively. The HPLC chromatograms after the analysis of the products obtained with flow chemistry and in batch mode are shown in Figure 4a,b, respectively.

This difference in reaction rate could be attributed to mass transfer limitations, since the nitration of 2,4-DNT could be considered as a biphasic reaction. Particularly, the organic phase consists of a melted mixture of DNT/TNT and the aqueous phase is a mixture of the used acids. Therefore, the distribution coefficients of reagents (DNT, HNO_3_, H_2_SO_4_) between the two phases significantly impact the kinetics of 2,4-DNT nitration (p. 314–318, [8]), [26]. H_2_SO_4_ practically exists only in aqueous phase, contrary to HNO_3_, which exhibits high solubility in the organic phase. Pure 2,4-DNT is soluble in the aqueous phase, but during the course of the reaction, and as the amount of TNT increases, the distribution coefficient shifts significantly to the organic phase. The organic phase, therefore, contains a high amount of 2,4-DNT and HNO_3_, but not H_2_SO_4_. This significantly affects not only the reaction rate, but most likely also the purity of the product since oxidation and side reactions can occur in absence of H_2_SO_4_. Linked to mass transfer limitations, the use of oleum in the reaction increases significantly the solubility of 2,4-DNT in the mineral acid phase and therefore facilitates the completion of the nitration, especially during the last stages of the reaction.

For these types of reactions, when mass transfer limitations can affect significantly the reaction progress, flow synthesis encompassing a rapid mixing reactor (contains chemical-resistant static mixers along its entire length) is superior to batch synthesis [27]. The intense mixing achieved using this kind of reactor is particularly effective for biphasic flow reactions due to the fact that the phases are prevented from separating. This results in a higher reaction rate and, in many cases, in a higher-purity product. The advantages of performing this kind of reaction by applying flow chemistry is highlighted also in the work published by Dumman et al. [28]. These authors investigated the nitration of a single aromatic substrate as an example of an exothermic two-phase liquid-liquid reaction, which reassembles in many regards the nitration of 2,4-DNT. The nitrating mixture in this reaction consisted of concentrated sulfuric and nitric acid. The reactor used was a capillary-microreactor suitable for biphasic reactions. These authors concluded that a rise in the conversion rate of the nitration reaction is linked to increased flow velocity, which enables vigorous mixing and enhances mass transfer. These findings are consistent with our results since a significant rise in the conversion rate was observed by performing the synthesis in flow compared to batch under the same conditions.

Another advantage of flow synthesis compared to batch-type reactions is the possibility of using elevated temperatures safely. Flow reactors facilitate the fast dissipation of heat (high surface-to-volume ratio) created during the highly exothermic reactions such as the mixing of sulfuric-nitric acid, nitration, or possible side reactions such as oxidation of nitro-aromatic compounds [29]. Heat transfer rate in flow reactors can be magnitudes of orders faster than in a batch reactor [17]. This prevents the hot-spot generation that can stimulate side reactions or runaway reactions to occur. It is reported that if the temperature applied for the DNT nitration in batch mode using an anhydrous nitration medium is higher than 120 °C, a runaway reaction is highly probable [6]. In the earlier methods of nitration, temperatures up to 120 °C were applied, but these methods were considered particularly hazardous due to the combination of concentrated acids and high temperature [8] (p. 391). Another factor that contributes to the enhanced safety of continuous systems is that the reacting volumes are much smaller than those applied in batch process, as stated also by Movsisyan et al. [15]. A comparison between the conventional batch processes and the reported synthesis is presented in Table 2.

The reagents HNO_3_ (65%) and H_2_SO_4_ (98%) are considerably cheaper than the fuming HNO_3_ (>90%) and oleum (>20% SO_3_) usually applied in batch methods. On the other hand, the larger excess of H_2_SO_4_ (98%) used compared to the batch methods could be considered as the main drawback of the flow chemistry method in terms of upscaling. A rudimentary economical assessment of the current flow chemistry method compared to the cost of the batch method in terms of the cost of reagents needed for the 2,4-DNT-to-TNT conversion (1 mole) is reported in Table 3 and Table 4. A number of batch methods for the third nitration step are described in the literature and reaction times vary from one to several hours and the DNT to nitric acid molar ratio varies from 2 to 3. The molar ratio of oleum to 2,4-DNT is around 5 (it can vary according to method) [7,8,12].

The cost comparison in Table 3 and Table 4 is of course for laboratory-scale production. A thorough economic assessment is beyond the scope of this paper.

### 2.4. NMR Analysis of the Produced TNT

As described in Section 2.2, high-purity TNT was achieved in several experiments. The TNT samples obtained at optimum conditions were also analyzed by ^1^H NMR in order to confirm the conclusions of HPLC analysis and further investigate the presence of any impurities not detectable by the applied HPLC-DAD method.

**Figure 5:**
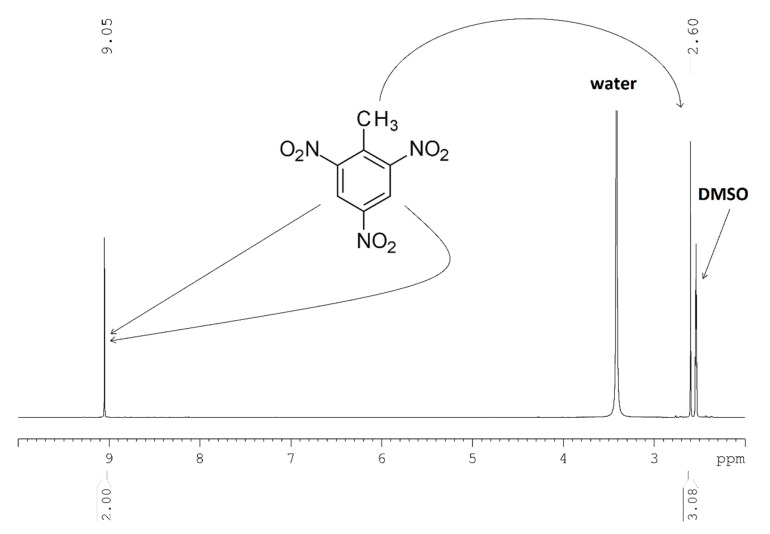
^1^H NMR spectrum of the synthesized TNT using flow chemistry in DMSO-d_6_.

Hence, it was important to investigate whether longer residence times or higher temperatures would result in the formation of by-products at certain HNO_3_:DNT molar ratios. Examples of such by-products include 2,4,6-trinitrobenzoic acid, 1,3,5-trinitrobenzene, and hydroxy-2,4,6-trinitrobenzoic acid [8] (pp. 300, 338). The NMR analysis was performed as described in Section 3.2.4. As illustrated in Figure 5, the ^1^H NMR spectrum consists of a singlet at 9.05 ppm corresponding to the aromatic protons of TNT and a singlet at 2.60 ppm corresponding to the methyl group of TNT. The integrals of the singlets showed a ratio of 2:3, as they are attributed to the two aromatic (2H) and the three aliphatic protons (3H), respectively (Figure 5). The additional signals in the aromatic region of the spectrum had a poor signal-to-noise ratio, which was below the limit of detection and quantification. This was consistent for all TNT preparations and confirms their purity.

## 3. Materials and Methods

### 3.1. Reagents and Equipment

All chemicals were of the highest quality available (i.e., with a negligible amount of impurities). 2,4-DNT, HNO_3_ 65%, H_2_SO_4_ 98%, dichloromethane, chloroform, and dimethyl sulfoxide-d₆ (99.9% D) were purchased from Sigma Aldrich (Overijse, Belgium), 2,4,6-TNT standard solution 1 mg/mL in Acetonitrile (Cerilliant, Round Rock, TX, USA) and LC/MS grade acetonitrile were purchased from VWR (Leuven, Belgium). The flow chemistry system applied for synthesis was Vapourtec RS-200 equipped with high acid resistant pumps (Vapourtec, Bury Saint Edmunds, UK), 20 mL large diameter (3 mm) tubular reactor for rapid mixing, and an automated fraction collector. The system configuration also included a peristaltic pump Gilson Miniplus 3.

A handheld Raman device (FirstDefender RMX, ThermoFisher Scientific, Waltham, MA, USA) and ion mobility spectrometers (IMS), namely the Itemiser DX (Rapiscan, Salfords, UK); Ionscan 500 DT (Smiths Detection, London, UK) and QS-B200 (L3 Technologies, New York, NY, USA) were used for the preliminary evaluation of the DNT conversion to TNT. A high-performance liquid chromatography (HPLC) Agilent 1200 HPLC system equipped with a Synchronis C18 column (Thermo Scientific, Waltham, MA, USA) and a diode array detector (Agilent Technologies, Inc., Santa Clara, CA, USA) was used to estimate the conversion of 2,4-DNT to TNT during experiments. The ^1^H-NMR analyses of synthesized TNT samples were performed by using an Ascend 400 MHz NMR spectrometer (Bruker BioSpin GmbH, Rheinstetten, Germany) equipped with a BBI (Broadband Inverse) probe.

### 3.2. Methods

The experimental design approach used to study and optimize the conditions for the flow chemistry system, the conditions adopted for both flow chemistry and batch synthesis, and the analytical methods applied to characterize the obtained products are described in this section.

#### 3.2.1. Experimental Design

After initial screening, three factors were identified as those that could affect the purity of TNT, namely the HNO_3_:DNT molar ratio, residence time (or reaction time), and temperature of the reaction. A face-centered design, a particular type of central composite design [22,30], was chosen in order to describe the response surface and to find the optimum synthesis conditions. The limits of each factor were established taking into account preliminary experiments, stoichiometry of the reaction, as well as physical and technical limitations such as viscosity of the solvent, maximum allowed temperature in the reactor, and allowed flow rate by the system. The chosen levels are presented in Table 5.

In total, 16 experiments were performed: one for each combination of the three parameter’s levels plus two central points in order to improve the mathematical characteristics of the model [22]. The DoE study and evaluation was performed using the software MODDE from Umetrics, Sartorius.

#### 3.2.2. Flow Chemistry Configuration

For TNT flow chemistry synthesis, the RS-200 flow chemistry system described above was equipped with high acid resistant pumps that enable the use of strong acids. The flow rate range of the pumps used was 0.05–10.00 mL/min, while the reactor temperature ranged from room temperature to 150 °C. The temperature of the reactor was controlled by the flow chemistry system. In each experiment, the flow of each pump was adjusted according to the desired residence time and stoichiometric ratio of reagents. Residence time (*R_t_*) was calculated according to the following equation:

(1) $$R_{t}\left(\min\right)=\frac{R_{v}}{Q_{1}+Q_{2}}$$

where *R_v_* is the reactor volume in ml and *Q_1_* and *Q_2_* are the flow rate in mL/min of pumps 1 and 2, respectively.

The accuracy of the flow rate was verified by measuring the consumed volume of reagents in certain time intervals. Fluctuations of temperature ±3 °C were observed during the run of the experiments. The experimental conditions were fed into the Vapourtec Flow Commander™ (Suffolk, UK) for reaction set up and control. This software includes a “dispersion modelling” tool (it models the axial dispersion occurring within the reactors) that is applied to automatically predict the steady state part of the reaction mixture flow stream. This is particularly beneficial for sample collection, which was performed by using the automated fraction collector integrated in the flow chemistry system.

**Figure 6:**
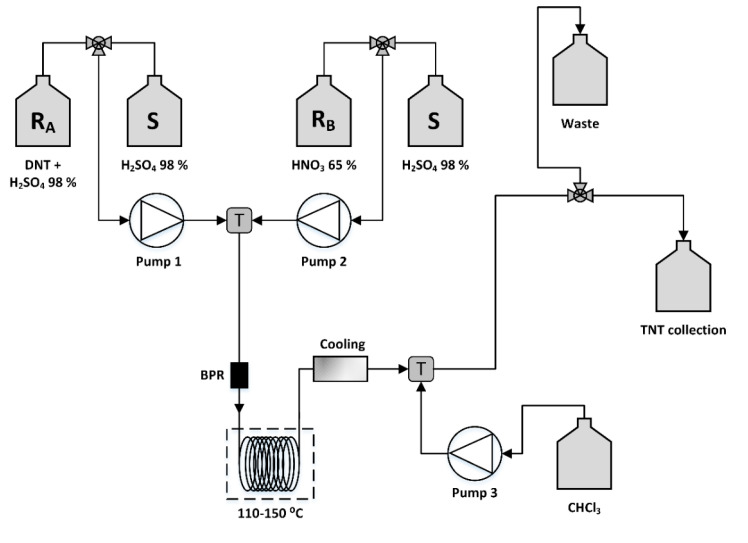
Configuration of the flow chemistry system used for TNT synthesis.

During method development, several experiments and modifications of the configuration of the flow chemistry system were performed in order to avoid precipitation of products and clogging of the system. Safety precautions were taken in order to minimize risks during the experiments. A distinct advantage of flow chemistry systems regarding safety is the automatic shut-down function of the apparatus if a rapidly increasing pressure is detected. Moreover, the system can also be manually stopped if an unforeseen temperature rise occurs. The configuration of the system used for TNT synthesis is depicted in Figure 6.

The solvent used for both lines was H_2_SO_4_ 98%, which also acts as the catalyst of the reaction in the nitrating mixture. The concentration of the reagents used were 0.56 M 2,4-DNT in H_2_SO_4_ 98% and HNO_3_ 65% (14 M). In the case of HNO_3_:DNT, the 1:1 molar ratio the concentration of 2,4-DNT increased to 1.1 M in order to adjust the flow of pump 2 (HNO_3_ 65%) at flow rates >0.05 mL/min, which is the minimum flow rate applicable to system pumps. As mentioned above, the feeding ratio of HNO_3_:DNT is controlled and adjusted by system pumps. Before starting the experiment, the pumps were thoroughly flushed with H_2_SO_4_ 98%. This was very important mainly for pump 1 since traces of water could promote DNT crystals formation and system clogging. The reagents were initially premixed in the T mixer before entering the 20 mL reactor where the reaction was performed at a controlled temperature. The reaction mixture was then cooled at room temperature and chloroform was added in the flow stream with the use of a peristaltic pump. In the collection vessel, dichloromethane and water were added in advance, and the purification was performed in a separatory funnel. The organic phase was washed with water, Na_2_CO_3_ 10% solution, and saturated NaCl solution and dried with Na_2_SO_4_. The solid product was obtained after evaporation of the solvent.

#### 3.2.3. Batch Synthesis

For the batch synthesis of TNT, a 250 mL glass round-bottom flask was used as the reactor vessel. First, 2.2 mL HNO_3_ 65% (≈30 mmol) was slowly added to the flask containing 1.82 g 2,4-DNT (≈10 mmol) already dissolved in 17.8 mL H_2_SO_4_. The flask was kept in an ice bath during the dropwise addition of nitric acid. When the addition was completed, the mixture was heated to the desired temperature. The applied HNO_3_:DNT molar ratio, reaction time, and the temperature were chosen taking into account the optimal TNT conversion rate and safety aspects. In particular, the following conditions were applied: 3:1 HNO_3_:DNT molar ratio, 20 min reaction time, and 130 °C reaction temperature. At the end of the reaction time, the flask was cooled down. The subsequent procedure of separating the product was the same as the one adopted with the flow chemistry approach.

#### 3.2.4. Analytical Techniques for Characterizing Reaction Products

A quick evaluation of the efficiency of applied processes during preliminary experiments was performed with a Raman device using an incident wavelength of 785 nm and ion mobility spectrometers (IMS). The handheld Raman device is equipped with a dedicated software (version 4.4.1) that allows measuring Raman spectra at various measurement conditions and can identify the sample by comparing its spectrum with the library spectra. The software enables setting laser power and measurement time. A laser power of 250 mW was applied to measure solid TNT obtained by flow chemistry with a resolution of 7 to 10.5 cm^−1^ and a spectral range of 250 to 2875 cm^−1^. An acquisition delay was applied to all the measurements; this feature in which the laser is turned on after a time delay allows the user to get away from the sample being measured before the laser is turned on in case there is a detonation. Raman shifts and relative intensity of Raman bands of TNT were in agreement with previously reported Raman spectra [31,32,33] indicating that the flow chemistry process allows production of pure TNT. The measurements were performed in triplicate.

IMS are widely deployed for on-site detection of traces of explosives. In IMS, vapor samples are ionized at atmospheric pressure and those ions are characterized by their gas phase mobilities in a weak electric field. One microliter of TNT solution in acetonitrile (about 0.1 mg/mL) was deposited onto the IMS’s swabs. After solvent evaporation, the swabs were inserted into the thermal desorber of the IMS devices and the analysis started automatically. IMS trigger alarms due to formed TNT ions that traverse the drift tube to reach the detector. The TNT solution was tested by at least three repeated measurements on each of the above mentioned IMS devices.

Assessment of the conversion rate of 2,4-DNT to TNT during process optimization was done with the HPLC system described above. Isocratic separation of 2,4-DNT and TNT was performed by injecting 10 μL of solutions of about 0.1 mg/mL in acetonitrile. The mobile phase was acetonitrile/H_2_O 50:50 and a flow rate of 1.3 mL/min. The identification of 2,4-DNT and TNT was based on retention time and UV spectra profile (190–400 nm). The retention times, resolution of peaks, and spectra profiles of analytes were determined after injection of standards and mixtures of pure compounds. The conversion rates were calculated based on the integrated peak areas of TNT and DNT at 254 nm, where 2,4-DNT and TNT demonstrate similar UV absorption. In order to estimate the conversion rate more precisely, the response factors of the two compounds at 254 nm were calculated by preparing and analyzing a standard containing the two molecules at equal concentration.

The HPLC analysis conclusions of TNT samples were further confirmed by ^1^H NMR. TNT samples were dissolved in DMSO-d_6_ and the ^1^H NMR spectra of different preparations were acquired. In all preparations the signals of TNT were identified.

## 4. Conclusions

The feasibility of performing nitration of 2,4-DNT to 2,4,6-TNT using a flow chemistry approach was demonstrated. The main advantages of the flow chemistry approach include the use of safer reagents (H_2_SO_4_ 98%, HNO_3_ 65% instead of oleum and fuming HNO_3_) and shorter reaction times (20-30 min). Moreover, the risk of runaway reactions is minimized as the mixing steps take place in the reactor under continuous flow conditions. The influence of key parameters, such as HNO_3_:DNT molar ratio, residence time, and process temperature were investigated and optimized by applying a design-of-experiments approach. We note the possibility of obtaining a high conversion rate from 2,4-DNT to TNT (>99%) in only 20 min. This significant improvement of reaction performance can be attributed to the use of a flow chemistry set up, which includes the rapid mixing in the reactor that facilitates enhanced mass transfer during the course of the reaction. It is possible to safely apply elevated temperatures due to the fast dissipation of heat, which would not be possible with the batch methods due to high risk of a runaway reaction. By comparison, in similar conditions, the conversion rate of 2,4-DNT to TNT in a batch type reaction did not exceed 58%. Although a thorough economic assessment is beyond the scope of this paper, a provisional cost assessment of reagents indicates the flow chemistry approach might be favorable.

We consider the approach described in this article as fit for purpose to safely produce high-purity, trace-level solutions that are needed, for example, by aviation security inspectors to verify that explosives trace detection equipment (ETD) used at airports continue to perform according to the specifications laid down in the EU Commission Implemented Regulation 2015/1998 [13] and the associated implementing legislation.

## Figures and Tables

**Table 1:** Reaction conditions and obtained conversion rates of the experiments performed according to the design of experiments (DoE) approach.

Experiment	Factors			Response
	HNO_3_:DNT Molar Ratio	Residence Time (min)	Temperature (°C)	Conversion Rate (%)
1	1	10	110	21.4
2	5	10	110	66.0
3	1	10	150	55.8
4	5	10	150	99.1 *
5	1	30	110	47.4
6	5	30	110	89.1
7	1	30	150	78.0
8	5	30	150	100.0 *
9	1	20	130	62.3
10	5	20	130	99.0 *
11	3	20	110	84.4
12	3	20	150	100.0 *
13	3	10	130	97.0
14	3	30	130	100.0 *
15	3	20	130	99.5 *
16	3	20	130	99.9 *

* Experiments that yielded a conversion rate ≥99% (calculated according to the purity of the final product as determined by HPLC-DAD).

**Table 2:** Comparison of the conventional batch process and flow synthesis for the third nitration step for TNT synthesis.

	Flow Chemistry	Batch
Time	Faster (residence time 10–30 min)	Longer reaction time > 1 h
Safety	Reaction performed safely up to 150 °C (enhanced heat transfer, only small fragment of the reaction mixture is present in the reactor at any time).	Applied temperatures, typically 90–115 °C (slower heat transfer, all the reaction mixture is loaded in the reactor). Higher temperatures for this nitration step are considered particularly hazardous.
	Homogenous and reproducible mixing results in better control of reaction parameters. Low probability of a runaway reaction.	Less homogenous mixing. Higher probability for hot-spot generation and a runaway reaction.
	Less hazardous reagents required (HNO_3_ 65%, H_2_SO_4_ 98%)	Fuming HNO_3_ > 98% (a particularly hazardous reagent to handle) and oleum (SO_3_ > 20%) are usually applied
Reproducibility	Flow synthesis processes are easy to reproduce and have homogenous mixing in microreactors. Automated and accurate control of reaction parameters.	More difficult to accurately control the reaction parameters. Non-homogenous mixing could result in lower conversion rates or higher amounts of each by-product.
Scaling up	The scaling up in flow can be easier and without additional hazards as reagent streams continuously pump into the reactor and product leaves the reactor as a continuous stream [15]. Scaling up can be performed by running the process for a longer time. The use of larger-volume reactors needs to be investigated.	Large scale commercial production exists.

**Table 3:** Approximate cost of the reagents needed for the conversion of 1 mole 2,4-DNT to TNT using the conventional batch method.

Reagent	Moles	Amount (mL)	VWR Code	Price * (EUR/l)	Cost (EUR)
HNO_3_ (≥90%)	2–3	90–140	ACRO270620010	211	19–30
Oleum (20% SO_3_)	5	250	30736-1L	192	48
				Total	approx. 67–78

* The calculated amount was based on the purchase of the largest available packaging on the website of chemical supplier VWR for Belgium (be.vwr.com).

**Table 4:** Approximate cost of the reagents needed for the conversion of 1 mole 2,4-DNT to TNT using the described flow chemistry synthesis.

Reagent	Moles	Amount (mL)	VWR Code	Price * (EUR/l)	Cost (EUR)
HNO_3_ (65%)	3	200	1.00443.9025	12.5	2.5
H_2_SO_4_ (98%)	solvent for DNT	1800	1.12080.9025	14	25
Chloroform **	extraction	1000	(ACRO 158210250) or 22720.462	11–24	11–24
				Total	approx. 39–52

* The calculated amount was based on the purchase of the largest available packaging on the website of chemical supplier VWR for Belgium (be.vwr.com); ** The lower price of chloroform is due to the possibility of using technical grade chloroform for extraction with negligible impact to the final product quality.

**Table 5:** Limit levels of and central points of the three factors considered in the experimental design.

Factor	Level		
	−1	0	+1
HNO_3_:DNT molar ratio	1	3	5
Residence time (min)	10	20	30
Temperature (°C)	110	130	150
